# Personality and Well-Being Across and Within Relationship Status

**DOI:** 10.1177/01461672231225571

**Published:** 2024-02-07

**Authors:** Elaine Hoan, Geoff MacDonald

**Affiliations:** 1 University of Toronto, Ontario, Canada

**Keywords:** single, romantic relationships, personality, well-being

## Abstract

Trends of increasing singlehood call for understanding of well-being correlates across and within relationship status. While personality is a major predictor of well-being, descriptive trait profiles of singles have not been developed. In the present research (*N* = 1,811; 53% men; *M*_age_ = 29), single and partnered individuals completed measures of personality and well-being, including life, relationship status, and sexual satisfaction. Results revealed effects whereby single individuals were lower in extraversion and conscientiousness but higher in neuroticism. Additional facet analyses showed that singles were lower across all extraversion facets, but specifically lower in productiveness (conscientiousness facet) and higher in depression (neuroticism facet). Largely, personality was associated with well-being similarly for single and partnered people. Furthermore, relationship status accounted for variance in well-being above and beyond personality traits. Our results suggest individual differences in personality could play an important role in understanding well-being’s link with relationship status.

## Introduction

Around the world, marriage rates are declining and the number of individuals living alone is at an all time high (Kislev, 2019; Organisation for Economic Co-Operation and Development, 2020). Some data suggest that a substantial proportion of unmarried individuals do not have a romantic partner (Tang et al., 2019) pointing to an overall rise in the number of singles (defined herein as individuals who are not in a serious romantic relationship). These rising trends in singlehood have sparked an increase in attempts to understand singles’ well-being within the public and academic spheres. Existing literature comparing the well-being of singles with that of individuals in romantic relationships has suggested a difference such that singles are, on average, lower on many well-being indicators than individuals in relationships (Luhmann et al., 2012). However, a growing body of research examining singles’ well-being has challenged these past findings, suggesting that the differences in happiness between singles and partnered people are small or even nonexistent, at least for subgroups of singles such as the never married (DePaulo & Morris, 2005; Greitemeyer, 2009). These mixed results have sparked a proliferation of research aimed at better understanding the factors associated with happiness in singlehood in a fashion that does not fall victim to societal stereotypes of single people as necessarily unhappy.

An important question when examining well-being is the role of personality, as individual differences along personality dimensions are reliable predictors of a variety of indicators of well-being across the life span (Lucas & Diener, 2000). One dominant framework for characterizing individual differences in personality is the five-factor model (FFM) that posits that five factors underlie personality (i.e., Extraversion, Agreeableness, Conscientiousness, Neuroticism, Openness to Experience; McCrae & John, 1992). In particular, extraversion and neuroticism appear to be strong predictors of well-being (Anglim et al., 2020). Given the reliable association between personality and well-being, identifying links between personality and relationship status could broaden our understanding of well-being both in singlehood and within relationships.

Notably, some existing research has pointed to personality differences that might be present across relationship status (herein operationalized as single vs. partnered). First, multiple studies have suggested that extraversion may be related to relationship status. Some work suggests that extraversion may be negatively associated with the likelihood of entering a relationship. In one study, Apostolou and Tsangari (2022) asked university students to report whether they were single due to difficulties attracting a partner or whether they were single by choice. They found that singles who faced difficulties with entering a relationship, but who wanted to obtain one, were lower in extraversion than already partnered individuals. Indeed, data collected from undergraduates between October 2020 and April 2021 during the COVID-19 pandemic when lockdown restrictions were in place (Chopik et al., 2023) found that extraverted people were 10% to 26% more likely to enter a new relationship during this time. Other work has examined whether entering a relationship may cause changes in extraversion. For example, one longitudinal study asked participants to record the occurrence of several life events, including entering a new romantic relationship (Dugan et al., 2023). Results revealed that extraversion increased after dating someone new (see also Neyer & Lehnart, 2007). However, a meta-analysis by Bühler et al. (2024) showed no effect of relationship status transition on extraversion. Thus, there is some evidence that extraverts may be more likely to enter relationships and more mixed evidence regarding whether people who enter relationships may experience changes in extraversion. Overall, the existing research does not clearly point to whether single and partnered individuals differ in average levels of extraversion.

Some research suggests single and partnered individuals may also differ in average levels of neuroticism. In one study, longitudinal evidence across a 4-year period showed that German adults who were initially single, but developed a romantic relationship by the end of the study period were less neurotic than participants who stayed single (Neyer & Asendorpf, 2001). Neuroticism is also associated with a number of factors linked to singlehood, such as depression, anxiety, and anxious attachment. Although neuroticism is not a necessary prerequisite for mood and anxiety disorders, neuroticism is strongly correlated with symptoms of depression and anxiety (Jylhä & Isometsä, 2006). One study found that greater depression and general anxiety was more prevalent among long-term singles than in coupled participants (Schachner et al., 2008). In addition, neuroticism is strongly associated with greater attachment insecurity (Crawford et al., 2007). Previous work has shown that attachment anxiety and attachment avoidance were more prevalent among long-term singles than coupled people (Pepping et al., 2018). Anxious attachment in particular has been shown to be associated with particularly strong desire for a romantic partner (MacDonald & Park, 2022) and a higher likelihood of settling for less appealing partners and less satisfying relationships due to greater fear of being single (Spielmann et al., 2013). Although these data suggest that individuals higher in anxious attachment may be more likely to be in relationships as a result of their fears of being single, they may also experience less stability in their relationships (McNelis & Segrin, 2019), which could decrease their likelihood of being in a relationship at any given time. However, the Bühler et al. (2024) meta-analysis did not evidence significant changes in neuroticism related to relationship status transition. Overall, then, the existing data cannot directly or clearly address whether singles and partnered individuals are likely to differ in their levels of neuroticism.

When it comes to the other FFM traits of Conscientiousness, Agreeableness, and Openness, some research shows that these traits are associated with well-being outcomes within relationships and within singlehood, but whether singles and coupled people differ in these traits is less clear. Within relationships, self-reported conscientiousness has been found to be linked to greater self-reported relationship quality while self-reported agreeableness was associated with greater relationship quality as rated by observers (Holland & Roisman, 2008). In addition, research showed that openness to experience was associated with greater relationship length in partnered individuals (Shaver & Brennan, 1992) and a preference for singlehood in singles (Apostolou & Tsangari, 2022). Moreover, Chopik and colleagues’ (2023) study conducted during the COVID-19 pandemic also showed that conscientious people were 15% to 17% less likely to start a new relationship during lockdown. In addition, Bühler et al. (2024) provided meta-analytic evidence that conscientiousness increases after entering a relationship. Despite these links between conscientiousness, agreeableness, openness, and well-being outcomes across relationships and singlehood, whether singles and coupled people differ on average in these traits cannot be concluded based on existing research.

Importantly, other research examining personality differences by relationship status found no evidence of average differences on any trait. In one study, singles and coupled people were asked to rate the personality traits of themselves and each other using single-item personality measures (Greitemeyer, 2009). Results demonstrated that singles and coupled people did not significantly differ on any measured personality traits. In addition, work by Shaver and Brennan (1992) also showed no correlation between one’s relationship status of involvement in a relationship and any of the big five traits using the NEO-Pi scales. Thus, these contradictory findings reinforce that whether personality traits differ by relationship status remains unclear.

An important issue in considering personality’s role in the link between relationship status and well-being is what indicators are particularly pertinent to understanding well-being across relationship status. One important indicator of overall well-being is life satisfaction, defined as one’s global assessment of quality of life (Shin & Johnson, 1978). Diener et al. (1985) proposed that life satisfaction is one of three primary components of well-being. Past research has demonstrated a difference in life satisfaction between partnered individuals and single individuals whereby partnered individuals appear happier on average (Adamczyk & Segrin, 2015; Dush & Amato, 2005). These findings suggest that relationship status may be importantly tied to life satisfaction. Another important indicator of well-being that may be more domain-specific is sexual satisfaction, the fulfillment of sexual needs. One systematic review showed that sexual satisfaction is associated with overall well-being, including a host of physical and psychological health factors (Sánchez-Fuentes et al., 2014). Notably, sexual satisfaction appears particularly linked to intimate relationships. Although singles who report higher sexual satisfaction also tend to have higher overall well-being (Kislev, 2021; Park et al., 2021), individuals in romantic relationships are considerably higher in sexual satisfaction on average presumably due to more frequent opportunities for partnered sexual activity (Park & MacDonald, 2022). Furthermore, satisfaction with one’s current relationship status as a single or partnered individual has been shown to be more strongly associated with well-being than other sociodemographic factors and even relationship status itself (Lehmann et al., 2015). As such, measuring subjective relationship status satisfaction is important for understanding well-being in the context of relationship status research. Overall, life satisfaction, sexual satisfaction, and relationship status satisfaction all appear to have important associations with well-being across relationship status and thus are valuable to examine in the context of examining the role of personality in understanding correlates of relationship status.

The goal of the current research was first to provide a comprehensive examination of the links between personality traits and relationship status. Furthermore, we aimed to understand how the associations between each personality trait and well-being differ depending on relationship status. That is, do any of the big five personality traits predict well-being indicators differently for single and coupled individuals? Finally, we examined whether any personality differences across relationship status could account for differences in well-being, or whether there is more to the link between relationship status and well-being than the personality characteristics of single and partnered individuals. To accomplish these aims, we collected self-reported personality data from one online sample to conduct exploratory analyses followed by a second online sample to examine replicability of our initial results. Importantly, because the following analyses were exploratory, no hypotheses were preregistered.

## Method

### Participants

All participants were recruited online through Prolific. Materials and data are available at https://osf.io/pa7zu/?view_only=a09d2c20996d4c7d904409a04e3b9bcb. To be eligible for the study, participants were required to be between the ages of 20 and 59 and be single for at least 6 months or in a relationship for at least 6 months. We conducted a power analysis to determine a sample size with at least 80% power. Based on the results of pilot data,^1^ we expected a minimum effect size of *partial r*^2^ = .10. The power analysis yielded a minimum sample size of 782 participants. To account for an expected dropout rate of 10% (Peer et al., 2017), we recruited an additional 78 participants.

Within the first sample, a total of 860 participants completed the study, which surpassed the minimum required sample size to achieve at least 80% power. We excluded 19 participants who did not consent to the study at the start, 20 participants who failed our attention checks, 55 participants who failed to complete the study, and six participants who reported that they did not respond honestly to our measures. The final sample consisted of a total of 819 participants (*M*_age_ = 29.49, *SD*_age_ = 9.48). Of this total, 402 participants reported currently being in a relationship (233 men, 200 women, eight nonbinary, one who preferred not to disclose; *M*_age_ = 29.43, *SD*_age_ = 8.85) while 358 reported being single (229 men, 150 women, nine nonbinary, three who preferred not to disclose; *M*_age_ = 29.22, *SD*_age_ = 10.00). Most of the participants were White (*n* = 585), 91 were Black, 19 were Asian, 52 were Mixed, 23 were of another unlisted ethnicity, and 49 did not report their ethnicity. Participants’ nationalities were heterogeneous, but participants primarily came from the United Kingdom (*n* = 146), Portugal (*n* = 126), South Africa (*n* = 107), Poland (*n* = 99), Italy (*n* = 59), Mexico (*n* = 46), and 49 participants did not report their nationality. The average relationship length was 5 years 8 months (range = 6 months–38 years, *SD* = 6 years 6 months) while the average length of singlehood was 5 years 8 months (range = 6 months–51 years, *SD* = 8 years, 3 months).

In the second sample, we conducted a power analysis to determine a sample size with at least 80% power based on the results from Sample 1. We expected a minimum effect size of *partial r*^2^ = .02. The power analysis yielded a minimum sample size of 986 participants. To account for a dropout rate of approximately 10% (Peer et al., 2017), we recruited an additional 90 participants. A total of 1,076 participants completed the study that surpassed the minimum required sample size to achieve at least 80% power. We excluded 19 participants who did not consent to the study, 17 participants who failed our attention checks, 46 participants who failed to complete the study, and two participants who reported that they did not respond honestly to our measures. Our final sample size consisted of 992 participants (*M*_age_ = 29.15, *SD*_age_ = 8.79). Of this total, 515 participants reported currently being in a relationship (247 men, 257 women, nine nonbinary, two who preferred not to disclose; *M*_age_ = 29.56, *SD*_age_ = 8.66) while 477 reported being single (252 men, 217 women, six nonbinary, two who preferred not to disclose; *M*_age_ = 28.71, *SD*_age_ = 8.92). The average relationship length was 5 years 6 months (range = 6 months–35 years, *SD* = 6 years 9 months) while the average length of singlehood was 4 years 8 months (range = 6 months–49 years, *SD* = 7 years 3 months). Most participants were White (*n* = 660), 135 were Hispanic, Latino, or Spanish, 129 were Black, 34 were Asian, 24 Native Hawaiian, eight Middle Eastern, and two American Indian or Alaska Native. Similar to Study 1, participants in Study 2 had a variety of nationalities, including but not limited to Portugal (*n* = 156), the United Kingdom (*n* = 155), Poland (*n* = 128), South African (*n* = 126), Mexico (*n* = 100), and 12 did not report their nationality.

## Materials and Procedure

Among a larger set of questionnaires, all participants across both samples completed the following measures listed below. Measurement invariance across relationship status was examined for each scale and results demonstrated adequate to good model fit with configural invariance, metric invariance, and scalar invariance across groups (see Supplementary Material A for full results).

### Personality

The Big Five Inventory–2 (BFI-2; Soto & John, 2017) is a 60-item scale that includes 12 items for each of the big five traits (see Supplementary Material B for facet reliabilities). These traits are extraversion (Sample 1: α = .86; Sample 2: α = .86), where the subscales include the facets of sociability (e.g., “Is outgoing, sociable”), assertiveness (e.g., “Is dominant, acts as a leader”), and energy level (e.g., “Is full of energy”); agreeableness (Sample 1: α = .79; Sample 2: α = .79) which includes the compassion (e.g., “Is compassionate, has a soft heart”), respectfulness (e.g., “Is polite, courteous to others”), and trust (e.g., “Assumes the best about people”) facets; conscientiousness (Sample 1: α = .86; Sample 2: α = .86) which includes the organization (e.g., “Keeps things neat and tidy”), productiveness (e.g., “Is efficient, gets things done”), and responsibility (e.g., “Is dependable, steady”) facets; neuroticism (Sample 2: α = .91) which includes the anxiety (e.g., Can be tense), depression (e.g., Often feels sad), and emotional volatility (e.g., “Is temperamental, gets emotional easily”) facets; as well as openness (Sample 1: α = .81; Sample 2: α = .80) including the intellectual curiosity (e.g., “Is complex, a deep thinker”), aesthetic sensitivity (e.g., “Values art and beauty”), and creative imagination (e.g., “Is curious about many different things”) facets. All items were rated on a 5-point scale ranging from 1 (*disagree strongly*) to 5 (*agree strongly*).

### Satisfaction With Relationship Status

Satisfaction with relationship status was assessed using the Satisfaction with Relationship Status Scale that consisted of four items (e.g., “How happy are you with your current status?”; Lehmann et al., 2015; Sample 1: α = .94; Sample 2: α = .93). Participants were asked to think about their own status, whether that was being single or in a relationship, when responding to the questions. All items were rated on a 4-point scale ranging from 1 (*not at all*) to 4 (*to a great extent*).

### Life Satisfaction

Life satisfaction was assessed using the Satisfaction with Life Scale that consists of five items (e.g., “In most ways my life is close to my ideal”; Diener et al., 1985; Sample 1: α = .91; Sample 2: α = .90). All items were rated on a 7-point scale ranging from 1 (*strongly disagree*) to 7 (*strongly agree*).

### Sexual Satisfaction

Sexual satisfaction was assessed using the Sexual Satisfaction Scale that consisted of four items (e.g., “I am satisfied with the sexual aspects of my life”; Park & MacDonald, 2022; Sample 1: α = .98; Sample 2: α = .98). All items were rated on a 7-point scale ranging from 1 (*not at all*) to 7 (*extremely*).^2^

## Results

All statistical analyses were conducted in R (R Core Team, 2021). Please see Supplementary Material C for all R packages used. Correlational analyses and descriptive statistics of the variables of interest can be found in Table 1.

**Table 1.** Correlations and Descriptive Statistics Between Variables in Sample 1 and Sample 2.

Variable	1	2	3	4	5	6	7	8
Sample 1								
1. Status Satisfaction	—	.38***	.51***	.13*	.17**	.19***	−.10*	.02
2. Life Satisfaction	.42***	—	.44***	.32***	.15*	.33***	−.43***	.07
3. Sexual Satisfaction	.54***	.37***	—	.24***	.13*	.17**	−.23***	.01
4. Extraversion	.04	.32***	.13*	—	.15*	.35***	−.39***	.29***
5. Agreeableness	.14*	.19**	.08	.15*	—	.34***	−.34***	.09
6. Conscientiousness	.07	.27***	.06	.36***	.33***	—	−.32***	.11
7. Neuroticism	−.32***	−.48***	−.18**	−.41***	−.34***	−.40***	—	−.04
8. Openness	.08	.08	.14*	.28***	.21***	.18**	−.00	—
*M* (Single)	2.54	3.32	2.50	2.90	3.66	3.34	3.17	3.80
*SD* (Single)	0.87	1.46	1.65	0.75	0.58	0.73	0.86	0.64
*M* (Partnered)	3.45	4.32	4.63	3.15	3.68	3.45	3.03	3.80
*SD* (Partnered)	0.63	1.44	1.69	0.72	0.56	0.68	0.87	0.61
Sample 2								
1. Status Satisfaction	—	.34***	.30***	.11*	.21***	.13*	−.10*	.12*
2. Life Satisfaction	.47***	—	.53***	.35***	.28***	.34***	−.37***	.12*
3. Sexual Satisfaction	.48***	.37***	—	.21***	.17**	.17**	-.17**	.11*
4. Extraversion	.17**	.43***	.23***	—	.16**	.37***	−.39***	.34***
5. Agreeableness	.17	.43**	.23	.23***	—	.34***	−.33***	.23***
6. Conscientiousness	.19***	.33***	.15***	.41***	.41***	—	−.39***	.13***
7. Neuroticism	−.32***	−.50***	−.24***	−.47***	−.47***	−.43***	—	−.13*
8. Openness	.13*	.02	.03	.24***	.24***	.15**	−.04	—
*M* (Single)	2.60	3.58	2.91	2.93	3.63	3.38	3.09	3.79
*SD* (Single)	0.83	1.47	1.78	0.73	0.57	0.69	0.84	0.58
*M* (Partnered)	3.44	4.43	4.93	3.11	3.70	3.47	2.98	3.77
*SD* (Partnered)	0.62	1.40	1.68	0.69	0.59	0.73	0.86	0.61

*Note.* Values below the diagonal indicate correlations among singles while values above the diagonal indicate correlations among partnered individuals. Descriptive statistics of each variable for single and partnered individuals are shown at the bottom. Significant correlations are bolded.

* *p* ≤ .05. **p ≤ .001. ***p ≤ .0001.

### Personality Differences Across Relationship Status

#### Big Five Domain Differences

As shown in the top half of Table 2, independent-samples *t*-tests were conducted to examine personality differences across relationship status. Results revealed a significant difference between partnered and single individuals’ levels of extraversion such that partnered individuals were higher in extraversion than single individuals. Moreover, individuals in relationships were also significantly higher in conscientiousness and lower in neuroticism compared with singles. No significant differences emerged between partnered individuals and singles with regard to their agreeableness or openness. As shown in the bottom half of Table 2, the pattern of findings from Sample 1 was similar to that of Sample 2. Once again, partnered individuals were significantly more extraverted, more conscientiousness, and lower in neuroticism than single individuals. Also, no significant differences emerged between partnered individuals and singles in their agreeableness or openness.

**Table 2.** Personality Differences Between Singles and Partnered Individuals in Sample 1 and Sample 2.

Sample 1:	Partnered		Single		*df*	*t*	*p*	95% CI		Cohen’s *d*
Personality	*M*	*SD*	*M*	*SD*				LB	UB	
Extraversion	3.15	0.72	2.90	0.75	750	4.74	**<.001**	4.59	5.1	.34
Agreeableness	3.68	0.57	3.66	0.58	752	.65	.52	0.60	0.76	.05
Conscientiousness	3.45	0.68	3.34	0.73	741	2.08	**<.05**	2.08	2.29	.15
Neuroticism	3.03	0.86	3.17	0.86	756	−2.25	**<.05**	1.99	2.27	−.16
Openness	3.80	0.61	3.80	0.64	747	0.00	.10	−0.09	0.09	.00
Sample 2:	Partnered		Single		*df*	*t*	*p*	95% CI		Cohen’s d
Personality	*M*	*SD*	*M*	*SD*				LB	UB	
Extraversion	3.12	0.73	2.93	0.69	975	4.10	**<.001**	4	4.37	.27
Agreeableness	3.70	0.57	3.63	0.59	987	1.90	.06	1.90	2.04	.12
Conscientiousness	3.47	0.69	3.38	0.73	990	2.13	**<.05**	2.12	2.32	.14
Neuroticism	2.98	0.84	3.09	0.86	987	−2.04	**<.05**	−1.82	−2.04	−.13
Openness	3.77	0.58	3.79	0.61	989	−.63	.53	−0.53	−0.69	−.04

*Note.* CI = confidence interval; LB = lower bound; UB = upper bound. Bolded *p*-values indicate significance levels below .05.

#### Facet Differences

To further assess relationship status differences across the 15 personality facets, we employed two-way 2 (relationship status: Single vs. Partnered) × 3 (facet: Facet 1, Facet 2, Facet 3) mixed analyses of variance (ANOVAs) with relationship status as the between person variable and the facets of each personality trait as the repeated measures variable for each trait domain in each sample (see Supplementary Material D and E for full ANOVA results and facet correlations).

Within the domain of extraversion, the interaction between relationship status and the facet levels of extraversion was nonsignificant in both Sample 1, *F*(2, 1494) = 1.81, *p* = .15, and Sample 2, *F*(2, 1942) = 0.51, *p* = .60, suggesting that the overall personality difference in extraversion between singles and partnered individuals does not differ significantly across the three extraversion facets.

Within the domain of agreeableness, the interaction between relationship status and the facet levels of agreeableness was significant in Sample 1, *F*(2, 1454) = 0.35, *p* = .02, but this interaction did not replicate in Sample 2, *F*(2, 1879) = 4.03, *p* = .70, indicating that personality differences in agreeableness do not reliably differ across the three facets.

A significant interaction did emerge between relationship status and the conscientiousness facets for Sample 1, *F*(2, 1472) = 3.12, *p* < .01, η^2^ = 0.01, and Sample 2, *F*(2, 1926) = 3.34, *p* < .05, η^2^ = 0.00, although the effect sizes were small in both samples. Simple main effect analyses with Bonferroni adjustments showed that partnered individuals were higher than single individuals in productiveness in Sample 1, *F*(1, 747) = 7.55, *p* < .05, η^2^ = 0.01 (*M*_Singles_ = 3.17, *SD* = 0.89, *M*_Partnered_ = 3.34, *SD* = 0.82), and Sample 2, *F*(1, 971) = 7.61, *p* < .01, η^2^ = 0.01 (*M*_Singles_ = 3.21, *SD* = 0.83, *M*_Partnered_ = 3.36, *SD* = 0.90). No significant differences emerged for the organization or responsibility facets.

For the neuroticism domain, a significant interaction between relationship status and the neuroticism facets also emerged across Sample 1, *F*(2, 1472) = 3.12, *p* < .05, η^2^ = 0.01, and Sample 2, *F*(2, 1925) = 5.75, *p* < .01, η^2^ = 0.01. Simple main effects demonstrated that singles were higher in depression in both Sample 1, *F*(1, 747) = 15.13, *p* < .001, η^2^ = 0.02 (*M*_Singles_ = 3.11, *SD* = 1.01, *M*_Partnered_ = 2.82, *SD* = 1.03), and Sample 2, *F*(1, 971) = 10.45, *p* < .01, η^2^ = 0.01 (*M*_Singles_ = 2.96, *SD* = 1.04, *M*_Partnered_ = 2.75, *SD* = 0.97), although the effect sizes were small. Meanwhile, no differences emerged for the anxiety and emotional volatility facets.

Finally, no significant interaction replicated between relationship status and the openness facets. While Sample 1 did not show a significant interaction, *F*(2, 1427) = 1.27, *p* = .28, Sample 2 did, *F*(2, 1867) = 5.21, *p* < .01.

### Do Personality Predictors of Well-Being Differ Across Relationship Status?

#### Personality Domain and Well-being Associations

To explore how relationship status moderates the personality and well-being link, a series of regression analyses were conducted on both samples examining each personality trait and relationship status as well as their interactions as predictors of each of the three well-being indicators. As shown in Table 3, the only interaction that replicated across the two samples was the interaction between neuroticism and relationship status in predicting relationship status satisfaction. To probe this interaction in Sample 1, simple slopes analyses were conducted, which demonstrated that higher levels of neuroticism were significantly associated with lower relationship status satisfaction for singles (*b* = –.32, *t* = –7.25, *p* < .001), but not for partnered individuals (*b* = –.07, *t* = –1.75, *p* = .08). Simple slopes analyses in Sample 2 similarly demonstrated that neuroticism was associated with lower relationship status satisfaction for singles (*b* = –.32, *t* = –8.08, *p* < .001), but not for partnered individuals (*b* = –.07, *t* = –1.86, *p* = .06). When conducting the same analyses with the other personality traits, no other interactions were significant (see Supplementary Material F for regression results for life satisfaction and sexual satisfaction outcomes).

**Table 3.** Personality Trait and Relationship Status Interactions Predicting Relationship Status Satisfaction Across Sample 1 and Sample 2.

Sample 1	Relationship status satisfaction				Sample 2	Relationship status satisfaction			
	β	*SE*	*t*	*p*		β	*SE*	*t*	*p*
IV: Extraversion	.09	.05	2.13	<.05	IV: Extraversion	.08	.05	2.11	<.05
Rel Status	−.40	.23	−3.05	<.001	Rel Status	−.64	.20	−5.33	<.0001
Interaction	−.12	.07	−0.90	.37	Interaction	.17	.07	1.39	.16
IV: Agreeableness	.12	.07	2.89	<.001	IV: Agreeableness	.14	.06	3.78	<.0001
Rel Status	−.55	.35	−2.79	<.001	Rel Status	−.20	.20	−1.10	.27
Interaction	.03	.09	0.17	.86	Interaction	−.30	.08	−1.68	.09
IV: Conscientiousness	.14	.05	3.22	<.001	IV: Conscientiousness	.10	.04	2.57	<.05
Rel Status	−.35	.27	−2.33	<.05	Rel Status	−.72	.23	−5.35	<.0001
Interaction	−.17	.08	−1.11	.27	Interaction	.24	.07	1.78	.07
IV: Neuroticism	−.07	.04	−1.75	.08	IV: Neuroticism	−.07	.04	−1.89	.06
Rel Status	−.07	.20	−0.64	.52	Rel Status	−.04	.17	−0.41	.68
Interaction	−.48	.06	−4.07	<.0001	Interaction	−.48	.05	−4.60	<.0001
IV: Openness	.01	.06	0.34	.74	IV: Openness	.09	.05	2.51	<.05
Rel Status	−.71	.34	−3.74	<.0001	Rel Status	−.59	.30	−3.32	<.0001
Interaction	.19	.09	1.00	.32	Interaction	.10	.08	0.53	.60

*Note.* Relationship status was a factor variable with partnered individuals coded as “1” and singles coded as “2.” Rel status = relationship status.

#### Personality Facet and Well-Being Associations

**Figure 1.**
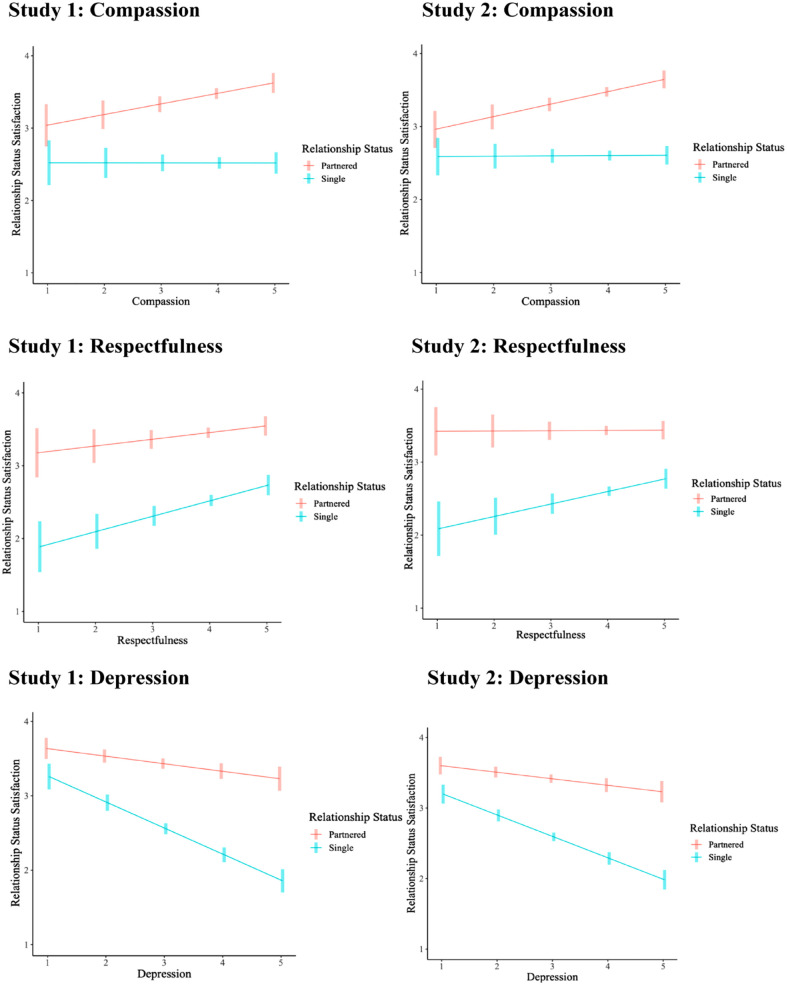
Plot of the Simple Slopes Analyses With Compassion, Respectfulness, Depression, and Relationship Status as Interacting Predictors. Study 1: Compassion; Study 2: Compassion; Study 1: Respectfulness; Study 2: Respectfulness; Study 1: Depression; Study 2: Depression. *Note.* Error bars represent standard errors.

To further examine how relationship status moderates the personality and well-being link, additional regression analyses were conducted with the personality facets. Specifically, we examined whether the facets for each personality domain interacted with relationship status to predict well-being. As shown in Figure 1, only agreeableness and neuroticism showed relationship status differences across facets (see Supplementary Material G for full regression results). When examining the relationship between relationship status satisfaction and the agreeableness facets, two-way interactions emerged for compassion (Sample 1: *b* = –.30, *t* = –3.41, *p* < .001; Sample 2: *b* = –.21, *t* = –2.72, *p* < .01), as well as respectfulness (Sample 1: *b* = .20, *t* = 2.06, *p* < .05; Sample 2: *b* = .17, *t* = 2.00, *p* < .05). Simple slopes analyses showed that compassion was associated with greater relationship status satisfaction for partnered individuals (Sample 1: *b* = .15, *t* = 2.86, *p* < .001; Sample 2: *b* = .17, *t* = 3.81, *p* < .001), but not for singles (Sample 1: *b* = –.00, *t* = –0.01, *p* = .99; Sample 2: *b* = .00, *t* = 0.10, *p* = .92). Moreover, respectfulness was associated with greater relationship status satisfaction for singles (Sample 1: *b* = .21, *t* = 3.62, *p* < .001; Sample 2: *b* = 0.17, *t* = 2.40, *p* < .05), but no significant link emerged for partnered individuals (Sample 1: *b* = .09, *t* = 1.63, *p* = .10; Sample 2: *b* = .01, *t* = 0.08, *p* = .93). Furthermore, when examining the link between relationship status satisfaction and neuroticism, a significant two-way interaction emerged with depression (Sample 1: *b* = –.24, *t* = –3.23, *p* < .01; Sample 2: *b* = –.22, *t* = –3.33, *p* < .001). Simple slopes analyses revealed that depression was associated with lower relationship status satisfaction for both singles and partnered individuals, but this relationship appeared stronger for singles (Sample 1: *b* = –.35, *t* = –9.28, *p* < .001; Sample 2: *b* = –.30, *t* = –9.78, *p* < .001) compared with partnered participants (Sample 1: *b* = –.10, *t* = –2.92, *p* < .001; Sample 2: *b* = –.09, *t* = –2.90, *p* < .001). All other facet and well-being associations did not differ significantly across relationship status.

### Is Relationship Status Associated With Well-Being Above and Beyond Personality?

Additional analyses were conducted to examine whether relationship status was a significant predictor of well-being above and beyond personality. To do so, we first examined whether well-being outcomes differed across relationship status by conducting a series of independent-samples *t*-tests.

#### Well-Being Differences Across Relationship Status

Across both samples, single individuals were shown to be significantly lower in terms of relationship status satisfaction, Sample 1: *t*(881) = –17.65, *p* < .0001, Sample 2: *t*(865) = –17.56, *p* < .0001; sexual satisfaction, Sample 1: *t*(974) = –18.16, *p* < .0001, Sample 2: *t*(962) = –18.26, *p* < .0001; and life satisfaction, Sample 1: *t*(975) = –9.28, *p* < .0001, Sample two: *t*(962) = –9.13, *p* < .0001.

### Relationship Status as a Predictor of Well-Being Controlling for Personality

To examine how much these differences across relationship status could be accounted for by personality, we conducted hierarchical multivariate regression analyses in both Sample 1 and Sample 2. In Step 1, the personality variables found to significantly differ across relationship status (extraversion, conscientiousness, and neuroticism) were entered simultaneously to predict relationship status satisfaction, sexual satisfaction, and life satisfaction. Multivariate regression was employed to account for related variance across the three well-being outcomes. In Step 2, relationship status was added as an additional predictor of the well-being outcomes to examine if it could account for variance beyond that accounted for by the personality variables.

Results demonstrated that adding relationship status resulted in a significant improvement in model fit, Sample 1: *F*(3, 753) = 125.32, *p* < .0001; Sample 2: *F*(3, 966) = 139.81, *p* < .0001. When only the personality variables were entered into the model in Step 1, they accounted for 5% to 6% of variance in relationship status satisfaction and 7% to 8% of variance in sexual satisfaction. For life satisfaction, the personality variables accounted for 25% of variance in both samples. However, in Step 2, there were overall increases in explained variance once relationship status was entered. For relationship status satisfaction, relationship status accounted for an additional 26% of variance in Sample 1 and 22% of variance in Sample 2 (Table 4). For sexual satisfaction, adding in relationship status resulted in a 25% increase in variance explained for Sample 1 and an additional 23% of variance accounted for in Sample 2. Whereas for life satisfaction, relationship status accounted for an additional 7% of variance in Sample 1 and 6% in Sample 2. Thus, in Step 2, the largest increases in explained variance were observed for relationship status satisfaction and sexual satisfaction relative to life satisfaction. Overall, relationship status contributed to an increase in explained well-being variance beyond extraversion, conscientiousness, and neuroticism.

**Table 4.** Hierarchical Multivariate Regression Analysis Results With Personality and Relationship Status Predicting Well-Being Outcomes.

	Rel status satisfaction		Life satisfaction		Sexual satisfaction	
Variable	*B* [95% CI]	β	*B* [95% CI]	β	*B* [95% CI]	β
Sample 1: Hierarchical multivariate regression						
Step 1: Personality						
Extraversion	.07 [−.02, .16]	.06	.35 [.21, .50]	.17***	.48 [.28, .69]	.18***
Conscientiousness	.07 [−.02, .17]	.03	.27 [.12, .41]	.10***	.07 [−.14, .28]	.06
Neuroticism	−.19 [−.27, −.11]	−.08***	−.61 [−.73, −.49]	−.59***	−.31 [−.49, −.13]	−.18***
*R*^2^	.05	—	.25	—	.07	—
*F*	15.82***	—	86.31***	—	21.27***	—
Step 2: Status						
Status	−.91 [−1.01, −0.80]	−.30***	−.83 [−1.01, −0.65]	−.47***	−2.01[−2.25, −1.78]	−.51***
△*R*^2^	.26	—	.07	—	.25	—
△*F*	70.54	—	5.05	—	70.91	—
Sample 2: Hierarchical multivariate regression						
Step 1: Personality						
Extraversion	.04 [−.08, .17]	.04**	.37 [.18, .57]	.23***	.22 [−.07, .52]	.20***
Conscientiousness	.08 [−.05, .21]	.04*	.22 [−.01, .43]	.11***	.18 [−.13, .49]	.13
Neuroticism	−.10 [−.21, .02]	−.04***	−.54 [−.72, −.35]	−.48***	−.15 [−.41, .12]	−.14**
*R*^2^	.06	—	.25	—	.08	—
*F*	21.86***	—	111.1***	—	29.62***	—
Step 2: Status						
Status	−.77 [−0.94, −0.61]	−.27***	−.76 [−1.04, −0.47]	−.42***	−1.70 [−2.10, −1.31]	−.48***
△*R*^2^	.22	—	.06	—	.23	—
△*F*	75.72	—	−2.00	—	78.08	—

*Note.* Beta values are displayed for each personality predictor and relationship status. Rel Status Satisfaction = relationship status satisfaction; CI = confidence interval.

* *p* ≤ .05. **p ≤ .001. ***p ≤ .0001.

## Discussion

Overall, our study provides one of the first comprehensive, descriptive profiles of personality trait similarities and differences across single and partnered individuals. Our data revealed reliable, average differences on some Big Five personality traits across relationship status. The biggest effect to emerge was that of extraversion such that singles were lower on extraversion than partnered individuals, an effect that was consistent across the facets of sociability, assertiveness, and energy level. In addition, we found smaller effects such that lower conscientiousness (particularly productiveness) and higher neuroticism (particularly depression) were more characteristic of single than partnered individuals. No reliable differences were found across studies for agreeableness or openness.

In addition, our data suggested that neuroticism (particularly depression) was linked to lower satisfaction with relationship status for singles, but not for partnered individuals. Moreover, within the agreeableness domain, while relationship status satisfaction was positively linked to compassion for partnered individuals (but not singles), relationship status satisfaction was positively associated with respectfulness for singles (but not partnered participants). No other personality variable showed evidence of differential associations with any of the well-being indicators across relationship status. Although extraversion, conscientiousness, and neuroticism were observed to differ significantly across relationship status, our data showed that relationship status still accounted for significant variance in well-being above and beyond these personality variables. Importantly, although replicated across two samples, our analyses were entirely exploratory and thus our interpretations of the data below are post hoc.

The data for relationship status differences across facets of extraversion revealed that singles were less extraverted than partnered individuals overall. These data may provide context for understanding how low levels of extraversion could be linked to both voluntary and involuntary reasons for being single (Stein, 1975). On one hand, single individuals may choose singlehood as a means of prioritizing opportunities for independence and solitude. For instance, Neel and colleagues (2016) surveyed a sample of U.S. adults and found that singles were higher on independence motives, such as comfort with alone time, which these authors found were moderately associated with introversion. Other evidence has shown that solitude is also linked to introversion. For example, Lin et al. (2020) found that more introverted individuals reported a greater capacity for solitude and a greater ability to enjoy alone time. The greater capacity of those higher in introversion to enjoy solitude suggests a better ability to capitalize on alone time in a positive way. As such, single individuals may seek singlehood as a means to yield benefits from independence and solitude.

At the same time, more introverted individuals may also have fewer opportunities to begin romantic relationships. One study by Apostolou (2019) found that singlehood may be associated with a host of introverted behaviors that may create barriers to successful relationship initiation. In this study, Apostolou examined responses to an online forum asking men about their reasons for being single. An “introversion” category emerged through qualitative coding of the responses that included low sociality and engagement in solo hobbies as reasons for being single. That is, introverted individuals may create environments that offer less opportunity to begin romantic connections. Altogether, past studies suggest that more introverted people may exhibit motivations and behaviors contributing to both voluntary and involuntary singlehood that may account for the relatively strong relation between relationship status and extraversion.

Yet another possibility is that romantic relationships may have causal effects on increasing extraversion. Indeed, Dugan and colleagues (2023) found that their participants tended to become more extraverted after entering a relationship. It is plausible that relationships increase extraversion by promoting greater confidence, social support, and access to new social networks (Parks, 2007). In theory, there could be a cycle such that introverts are less likely to enter relationships, and by staying single are also less likely to demonstrate increases in extraversion that those in relationships experience, thus sustaining more stable singlehood (cf. Bühler et al., 2024).

Of note, our data also suggested that singles were lower in conscientiousness than partnered individuals, although this difference was smaller in magnitude. One reason for this finding could be that effective goal pursuit abilities associated with conscientious individuals could make them more likely to start and maintain relationships (John & Srivastava, 1999). Our results support this notion by demonstrating that partnered individuals were particularly higher in the productiveness facet, which is characterized by a strong work ethic and persistence toward goal pursuit (Soto & John, 2017). While not everyone possesses the goal of entering a relationship, a strong percentage of individuals do, particularly in the young to middle adulthood life stages encompassing the majority of our participants (Pew Research Center, 2020). Insofar as relationship attainment and maintenance is a goal for many people, the goal pursuit behaviors of individuals high in productiveness may increase their likelihood of successfully starting and maintaining relationships. Indeed, conscientious individuals are more likely to apply an achievement-orientation toward romantic relationships and engage in greater intimacy and commitment (Engel et al., 2002). Similarly, Reisz et al. (2013) asked participants to list personal goals and found that conscientiousness was associated with goals to maintain romantic relationships. Thus, conscientious individuals appear to engage with relationships such that they are motivated to enhance relationship success.

Furthermore, highly conscientious individuals may be less likely to engage in negative impulses, like infidelity, that could lead to relationship dissolution (and thus return to singlehood). For example, Isma and Turnip (2019) showed that conscientiousness was associated with more negative attitudes toward infidelity. Other work supports this link and demonstrates that higher conscientiousness is associated with a lower likelihood of engaging in infidelity, including flirting and extra-marital affairs (Buss & Shackelford, 1997). Cognitive correlates of conscientiousness, such as greater executive control (Petrican & Schimmack, 2008), may also play a role in relationship maintenance. For example, Pronk and colleagues (2011) demonstrated in an experimental paradigm that partnered participants with higher levels of executive control were less likely to flirt with confederates or express desires for meeting attractive others. Overall, conscientiousness, and productiveness in particular, may be associated with traits like goal pursuit strategies and lower infidelity, which could be helpful in the initiation and maintenance of relationships. These characteristics may be mechanisms through which conscientiousness is somewhat higher among coupled people compared with singles.

The conscientiousness findings are also in line with previous longitudinal work showing that, over the span of 4 years, being in or entering a relationship is associated with increases in conscientiousness (Neyer & Asendorpf, 2001). As such, another possibility is that relationships can increase conscientiousness. While some studies reveal little to no effect of romantic relationships on personality change (Asendorpf & Wilpers, 1998), other research shows that transitioning into a partnership coincides with increases in conscientiousness (Bühler et al., 2024; Neyer & Lehnart, 2007). Relationship maintenance may well require rehearsal of conscientious behaviors such as upholding promises and remembering important duties (Hill et al., 2014). Over time, engaging in these conscientious behaviors could theoretically lead to personality changes. Moreover, while not all romantic relationships are satisfying, greater relationship satisfaction is linked to greater self-regulation and more effective daily goal pursuit (Hofmann et al., 2015). As such, the types of relationships that are more likely to last (i.e., more satisfying ones) are particularly likely to contain the beneficial components that could facilitate greater productiveness.

Of course, singles have many other domains in their lives that may facilitate the development of productiveness. For example, career and educational domains also require the rehearsal of conscientious behaviors for success. Indeed, an examination of European Social Survey data revealed that unmarried participants tended to possess the highest levels of educational attainment (Kislev, 2019). Moreover, singles have been shown to more frequently extend support to family, neighbors, and friends (Sarkisian & Gerstel, 2016). Having a diverse number of care responsibilities could promote productive habits like actively staying connected to others while balancing personal responsibilities. Various activities that are part of single lives could thus certainly promote conscientious behaviors through a number of possible domains, including career, education, and care responsibilities. However, one thing that people in romantic relationships have in common is engagement in one typically motivating life domain (their romantic relationships; Pew Research Center, 2020) which may encourage them and hold them accountable in developing conscientious behaviors.

Our findings also demonstrated that singles were somewhat higher in neuroticism (particularly depression) than partnered individuals, although this effect was also relatively small in magnitude. Importantly, the nature of this relationship is unclear. One possibility is that depression symptoms, like sadness and low energy, make it more difficult to start a relationship. Individuals high in depression are more likely to have deficits in perceptual (e.g., lack of eye contact and attentiveness) and cognitive (e.g., negative interpretations of social interactions) social skills (Tse & Bond, 2004). As a result, individuals dealing with depressive symptoms who want to be partnered may experience difficulties in romantic relationship pursuit (Pe et al., 2016).

Depression could also interfere with relationship maintenance, given there exists a well-documented link between depressive symptoms and relationship dissatisfaction (Whitton & Kuryluk, 2012). Indeed, one prospective study examining couples in Norway found that emotional distress, including depressive symptoms, were a risk factor for future relationship dissolution (Røsand et al., 2014). Depression may also influence relationship stability (and thus relationship status) due to its demonstrated links with undermining perceptions of reward in a relationship (Kurdek, 1997), reduced intimacy and passion (Engel et al., 2002), and dysfunctional conflict management patterns (Brose et al., 2005). Altogether, extant work suggests that depression can create challenges for relationship maintenance (and thus higher odds of returning to singlehood) through a number of maladaptive behaviors and thought patterns.

On the contrary, being in a relationship could potentially lower one’s depression. Previous longitudinal evidence supports this claim. For example, one study by Roberson and colleagues (2018) examined data from a national U.S. sample collected from 1986 to 2011. They demonstrated more rapid decreases in depressive symptoms for participants who transitioned into new relationships compared with participants who remained in stable relationships. Similarly, Barr et al. (2016) showed that transitioning into satisfying partnerships was associated with reduced depressive symptoms over a 2-year period, even after controlling for sex, age, and income. There are a number of reasons why levels of depression might decrease in romantic relationships. First, greater opportunities for emotional support within romantic relationships could reduce experiences of depressive symptoms. For example, Joosten and colleagues’ (2022) study on Dutch couples revealed that greater perceived support from one’s partner coincided with decreased depressive symptoms over a 2-year period. Another study examining undergraduate couples found that greater perceived partner support, including understanding their partner’s feelings, was linked to lower depressive symptoms, and subsequently, greater relationship satisfaction (Cramer, 2004). As such, satisfying romantic relationships could reduce levels of depression through pathways like increased emotional support. The relationship status differences in depression within our findings could thus be due to the reduction of depression within romantic relationships.

Although some previous literature supports the finding that levels of depression may be higher among single individuals, perhaps what is more novel is the particularly weak link between depression and relationship status satisfaction for people in relationships. This finding is of interest given the well-documented link between depression and lower relationship satisfaction, a link supported by meta-analytic evidence from examining marital relationships (Whisman, 2001). More broadly, depression is partly characterized by having more negative perceptions in general (Beck, 1967). Thus, it is somewhat surprising on its face that even at higher levels of depression, individuals in relationships are relatively high in satisfaction with their relationship status. This suggests that individuals higher in depression may be relatively unhappy with the quality of their specific relationship but simultaneously prefer to be partnered than single. Although surprising at one level, this finding is consistent with Spielmann et al.’s (2013) research on fear of being single, a variable strongly associated with depression. Those who fear being single are more likely to remain in less satisfying relationships to avoid being single (Spielmann et al., 2013). That is, it may be particularly the aspect of depression that is tied to fears of singlehood that maintains relationship status satisfaction among these individuals who typically experience lower relationship satisfaction.

In addition to neuroticism, relationship status interactions with agreeableness facets also emerged. Compassion, defined as emotional concern for others’ well-being (Soto & John, 2017), was associated with greater relationship status satisfaction for partnered individuals, but not for singles. That is, being higher in compassion is associated with being more satisfied about being in a relationship, but not more satisfied about being single. This finding aligns with existing work showing that compassion toward one’s partner is associated with greater relationship satisfaction (Bolt et al., 2019). The reason for this relationship is unclear, but it is plausible that individuals high in compassion may derive fulfillment through caring for close others. Indeed, Hadden and colleagues (2014) found that compassionate goals, such as desires to be helpful, are associated with greater relatedness needs. Moreover, compassionate goals were found to predict greater relationship satisfaction in both partners. As such, for individuals high in compassion, romantic relationships could be gratifying by providing one particularly intimate avenue through which compassionate goals and relatedness needs are fulfilled.

In addition, our data showed that respectfulness, defined as being considerate of others’ preferences while inhibiting one’s own antagonistic impulses (Soto & John, 2017), was associated with greater relationship status satisfaction for singles, but not for partnered individuals. Although this effect replicated across studies, it was one of our weaker replicated effects, and our literature search turned up little that satisfactorily explained this pattern. Thus, we note this effect replicated but leave it to future research to determine the reliability and meaning of this finding.

For extraversion, conscientiousness, and openness, no well-being interactions with relationship status emerged. That is, the association between these three traits and well-being did not differ between singles and partnered individuals. Furthermore, no interactions with personality traits were found in predicting life or sexual satisfaction. These results suggest that the traits related to well-being in relationships are largely comparable with traits linked to well-being in singlehood. In this light, singlehood and relationships do not appear to be fundamentally different psychological experiences in terms of what personality traits are most likely to promote well-being.

Understanding personality differences across relationship status may be beneficial in shedding light on data suggesting higher average well-being for partnered versus single people. As such, we further examined the extent to which differences in well-being between single and partnered individuals could be accounted for by personality differences in extraversion, conscientiousness, and neuroticism (the Big Five traits that differed across relationship status). We first examined well-being differences across relationship status and found that single individuals demonstrated lower overall relationship status satisfaction, sexual satisfaction, and life satisfaction compared with partnered individuals. Our data revealed that personality accounted for some variance in relationship status satisfaction and sexual satisfaction, but accounted for relatively more variance in life satisfaction. Furthermore, our analyses suggested that relationship status explained significant variance beyond personality, most strongly for relationship status satisfaction and sexual satisfaction but to some extent for life satisfaction. These findings suggest that individual differences can partially account for the well-being and singlehood link. However, these data further suggest that personality alone does not fully account for well-being differences across relationship status. Thus, the link between relationship status and well-being may involve factors beyond the personality traits associated with likelihood of being single or in a relationship.

One reason why relationship status was particularly strongly associated with relationship status satisfaction and sexual satisfaction could be that these two well-being indicators are particularly linked to romantic relationships (Park et al., 2021). That is, to the extent that desire for romantic partnership is relatively normative (although certainly not universal; Horowitz et al., 2019), and to the extent that the majority of partnered sexual activity occurs within committed relationships (Park & MacDonald, 2022), relationship status satisfaction and sexual satisfaction appear strongly tied to being in a romantic partnership regardless of personality traits. Meanwhile, life satisfaction may be less strongly linked to the domain of partnership such that general personality becomes more important than relationship status. Consistent with past research (Anglim et al., 2020), we found that those who experienced greater life satisfaction were higher in extraversion and conscientiousness, but lower in neuroticism. As such, a significant portion of relationship status differences in life satisfaction appears attributable to personality traits linked with relationship status rather than as a direct result of being single or partnered.

Nevertheless, a notable amount of variance in life satisfaction did appear attributable to relationship status beyond personality. This finding is consistent with evidence that relationships generate greater happiness (at least for those who are motivated to seek them out); longitudinal research finds that entering romantic relationships is associated with increases in life satisfaction (Bühler et al., 2024). Another contributor to the effect of relationship status on well-being could be singlism (DePaulo & Morris, 2005), that is, stigma and discrimination against singles. For example, Girme et al. (2022) showed that singles’ reports of societal discrimination were associated with lower well-being. As such, negative treatment of singles could detrimentally affect life satisfaction in a manner that could plausibly account for some or all of the well-being differences across relationship status unattributable to personality.

Of course, the link between relationship status and well-being could also be a result of third variables. Socioeconomic status (SES) is linked to both life satisfaction and relationship status. Longitudinal evidence documented that couples with greater economic disparity showed greater declines in relationship satisfaction and life satisfaction (Keizer & Komter, 2015). These disparities could potentially affect relationship maintenance and lead to relationship dissolution and singlehood. Moreover, SES may affect one’s relationship status through mate selection. One experimental study showed that women are more likely to select potential partners with high SES to form long-term relationships (Greitemeyer, 2005). As such, having a higher SES could be beneficial in entering a relationship and thus influence one’s relationship status. Altogether, SES may be one of many potential third variables explaining the relationship between life satisfaction and relationship status. In general, more research on the impact of SES on singlehood is needed.

Overall, these results suggest that personality plays a role in various well-being indicators, but that relationship status is most strongly associated with more relationship-oriented well-being outcomes (i.e., relationship status satisfaction and sexual satisfaction). Meanwhile, personality appears to play a particularly strong (but not exclusive) role in accounting for variance in life satisfaction.

The main strength of this research was its strongly powered samples that allowed our data to provide a reliable, descriptive profile of personality traits among single and partnered individuals. Moreover, while many of our observed effect sizes were relatively small, the extraversion difference yielded a moderately large effect size. In addition, because of our two sample approaches, we were able to reliably examine replicability of the personality differences and interactions across relationship status. Our study also employed measures of personality and well-being outcomes with strong psychometric properties (Lehmann et al., 2015; Park & MacDonald, 2022) to maximize the reliability and validity of our findings. However, these results would benefit from future replications and extensions that address the various limitations of these data.

Perhaps the biggest limitation of this study is the cross-sectional, and consequently correlational, nature of our data. These data limit our ability to examine changes over time, and more importantly, our ability to draw causal conclusions. While these findings are helpful in providing novel between-group insights into how personality differs between single and partnered individuals on average, we cannot determine the extent to which personality traits such as introversion are a cause or consequence (or both) of singlehood, or even a third variable related coincidence. More work employing prospective longitudinal designs and/or quasi-experimental methods to examine the extent to which personality differences lead to changes in relationship status or vice-versa seems warranted.

Furthermore, our sample specifically included more long-term singles and long-term partnered individuals (minimum 6 months) to better ensure that we were not sampling individuals whose relationship status was unclear or somewhere in between single and partnered. However, focusing on these specific samples limits our ability to generalize to singles or partnered individuals whose relationship status duration is less than 6 months. For romantic relationships spanning 6 months or less, the role of personality may be overwhelmed by feelings of infatuation. For example, conscientiousness may not be particularly needed for behaviors like remembering important dates in early relationships when many people experience chronic salience of their new partner (Langeslag et al., 2013). Furthermore, personality may also not fully reveal itself within the first 6 months of singlehood. Some evidence shows that relationship dissolution can coincide with negative personality changes, particularly for men (Asselmann & Specht, 2020). Thus, the traits that could eventually facilitate healthy singlehood may be overshadowed by the grief of relationship dissolution. Therefore, future studies should examine the role of relationship status and personality on well-being in early stages of a relationship or singlehood.

Moreover, we focused on between-group differences in personality between singles and partnered individuals, but examined within-group variability to a lesser extent. However, heterogeneity in personality nonetheless exists within singles and partnered individuals. For example, Walsh et al. (2022) found 10 profiles of singles that differed in their levels of well-being as well as neuroticism and extraversion, highlighting the diversity in singles’ traits (see Girme et al., 2023 for a review). Although we examined the role of within-group variability in predicting well-being, important within-group factors like marital history were unaccounted for. Existing work has shown that never married individuals are happier than divorced or widowed individuals (DePaulo & Morris, 2005) while married individuals living together show higher well-being than unmarried cohabitors (Shapiro & Keyes, 2008), suggesting within-group variability among singles and partnered individuals. As such, future studies would benefit from integrating within-group factors, like marital history, into their examination of personality differences related to relationship status.

Finally, our sample comprised primarily Western participants. Although participants were sampled from a general online pool that includes individuals from non-Western countries, these samples were insufficient for between-country analyses. As a result, our findings are culture bound such that they generalize only to the cultural contexts examined. For example, in cultures where individual choice plays less of a role in marriages or relationships, such as in more collectivistic societies (e.g., MacDonald et al., 2012), personality traits may be less likely to predict relationship status and well-being. Thus, future studies could recruit more diverse samples and focus on cultural differences, especially given that cultural research in singlehood is a relatively unexplored domain (Girme et al., 2023).

In conclusion, our study sought to provide a descriptive profile of personality traits across relationship status. The present research advances the singlehood literature by providing a trait overview of singles and partnered individuals, at least within the cultural contexts from which our participants were drawn. Our data suggest that personality differs across relationship status and that these traits may partially explain the relationship status and well-being link, but there appears to be more to this relationship than Big 5 personality traits. Overall, the present studies extend previous research to shed light on individual differences across relationship status and provide future directions for understanding the well-being implications of these characteristics.

## Supplemental Material

sj-docx-1-psp-10.1177_01461672231225571 – Supplemental material for Personality and Well-Being Across and Within Relationship Status Supplemental material, sj-docx-1-psp-10.1177_01461672231225571 for Personality and Well-Being Across and Within Relationship Status by Elaine Hoan and Geoff MacDonald in Personality and Social Psychology Bulletin

## Notes

1. These pilot data were from another study (*N* = 795) examining singles’ well-being and time-use that included a brief personality measure for exploratory purposes. These results demonstrated small effect sizes when examining correlations between personality and well-being. However, the personality measure used was brief, did not capture facets, and ultimately deemed unsatisfactory with regard to internal consistency (Romero et al., 2012) and measurement quality that motivated the present study.

2. An additional question was erroneously included in this measure that states “I am satisfied with the sexual aspects of my life,” but this item was not included in the analyses since the item is not a part of the Sexual Satisfaction Scale.
