# Supplementary material for: Personality and Well-Being Across and Within Relationship Status
Source: Pers Soc Psychol Bull. 2024 Feb 7;51(9):1648–63. doi: 10.1177/01461672231225571 (PMC12276397; doi:10.1177/01461672231225571)
Supplement: sj-docx-1-psp-10.1177_01461672231225571 – Supplemental material for Personality and Well-Being Across and Within Relationship Status [file sj-docx-1-psp-10.1177_01461672231225571.docx]

**Supplementary Materials**

**Supplementary Material A: Measurement Invariance Results**

Measurement invariance across relationship status was examined to assess whether the pattern of loadings (i.e., configural invariance), the magnitude of the factor loadings (i.e., metric invariance), and the item intercepts (i.e., scalar invariance) varied across singles and partnered individuals. Confirmatory factor analyses (CFA) with maximum likelihood robust (MLR) for model parameter estimation were conducted for each measure. To evaluate goodness-of-fit, we calculated comparative fit Index (CFI) and root mean square error of approximation (RMSEA). To establish cut-offs for measurement invariance, we followed Choi et al.’s (2021) approach and determined noninvariance for RMSEA changes larger than 0.015 and CFI changes less than or equal to 0.01. Analyses were conducted in R using the cfa() function from the *lavaan* package (Rosseel, 2012).

**Personality*.*** We tested a three-factor structure of personality subfacets for each of the five domains measured by the 60-item Big Five Inventory-2 (BFI-2). We examined the traits at the subfacet level opposed to the overall five factor model (FFM) because CFAs have typically shown poor fit for the FFM (Martin et al., 2010).

***Extraversion.*** The configural model for extraversion showed adequate fit across relationship status in both Sample 1 and Sample 2, CFI > .90, RMSEA < .04. Metric invariance was then assessed by constraining the factor loadings to be equal across relationship status. The change in model fit was below the cut-off for rejecting invariance in both samples, (Sample 1: ΔCFI = .001, ΔRMSEA = .004; Sample 2: ΔCFI = .00, ΔRMSEA = .003). Finally, scalar invariance across groups was assessed by adding constraints on the intercepts. Once again, the change in model fit was below the cut-off for rejecting invariance (Sample 1: ΔCFI = .005, ΔRMSEA = .00; Sample 2: ΔCFI = -.001, ΔRMSEA = -.003).

***Agreeableness.*** The configural model for agreeableness showed adequate fit in both Sample 1 and Sample 2, CFI > .90, RMSEA < .08. The change in model fit was below the cut-off for rejecting invariance when constraining just the factor loadings to be equal across groups (Sample 1: ΔCFI = .001, ΔRMSEA = -.004; Sample 2: ΔCFI = .009, ΔRMSEA = -.001) and when additionally constraining the intercepts (Sample 1: ΔCFI = -.003, ΔRMSEA = -.002; Sample 2: ΔCFI = -.005, ΔRMSEA = -.002).

***Neuroticism.*** The configural model for neuroticism showed adequate fit in both Sample 1 and Sample 2, CFI > .90, RMSEA < .08. The change in model fit was below the cut-off for rejecting invariance when constraining the factor loadings to be equal across groups (Sample 1: ΔCFI = .001, ΔRMSEA = -.005; Sample 2: ΔCFI = .00, ΔRMSEA = -.005) and when constraining the intercepts (Sample 1: ΔCFI = -.003, ΔRMSEA = -.002; Sample 2: ΔCFI = -.001, ΔRMSEA = -.003).

***Conscientiousness.*** The configural model for conscientiousness showed adequate fit in Sample 1 and Sample 2, CFI > .90, RMSEA < .08. The model showed metric invariance across groups when constraining the factor loadings to be equal (Sample 1: ΔCFI = -.003, ΔRMSEA = -.003; Sample 2: ΔCFI = -.002, ΔRMSEA = -.003). When adding the constraint on the intercepts, once again the change in fit was below the predetermined cut-offs (Sample 1: ΔCFI = -.006, ΔRMSEA = -.001; Sample 2: ΔCFI = -.005, ΔRMSEA = -.002).

***Openness.*** The configural model for conscientiousness showed adequate fit in Sample 1 and Sample 2, CFI > .90, RMSEA < .08. The changes in model fit were below the cut-offs for determining noninvariance when constraining the factors loadings to be equal across groups (Sample 1: ΔCFI = -.001, ΔRMSEA = -.004; Sample 2: ΔCFI = -.001, ΔRMSEA = -.004) and when additionally constraining the intercepts (Sample 1: ΔCFI = -.003, ΔRMSEA = -.001; Sample 2: ΔCFI = -.009, ΔRMSEA = .00).

Overall, the BFI-2 demonstrated measurement invariance across groups for each trait suggesting that the assessment of personality did not differ across singles and partnered individuals.

**Satisfaction with Relationship Status.** We tested a one-factor structure of relationship status satisfaction which is in line with the original scale development (Lehmann et al., 2015). The configural model showed good fit indices in Sample 1, CFI > .95, RMSEA < .05, but adequate fit in Sample 2, CFI > .95, RMSEA < .06. When constraining the factors loadings to be equal across group to test for metric invariance, the changes in model fit did not exceed the predetermined cut-offs (Sample 1: ΔCFI = .001, ΔRMSEA = -.008; Sample 2: ΔCFI = -.001, ΔRMSEA = .001). When testing for scalar invariance, changes in model fit once again did not surpass the cut-offs after additionally constraining the intercepts, (Sample 1: ΔCFI = -.004, ΔRMSEA = -.001; Sample 2: ΔCFI = -.003, ΔRMSEA = .003). Overall, the Satisfaction with Relationship Status scale demonstrated measurement invariance across groups suggesting that the assessment of relationship status satisfaction did not differ across singles and partnered individuals.

**Life Satisfaction*.*** A one-factor structure of life satisfaction was tested which is in line with the development of the scale (Diener et al., 1985). The configural model of the one-factor structure showed good fit in Sample 1, CFI > .95, RMSEA < .08, but adequate fit for Sample 2, CFI >.95, RMSEA < .05. When constraining the factor loadings to examine metric invariance across relationship status, changes in model fit did not surpass the predetermined cut-offs for both samples (Sample 1: ΔCFI = -.001, ΔRMSEA = -.008; Sample 2: ΔCFI = .00, ΔRMSEA = -.012). Similarly, changes in model fit when additionally constraining the intercepts did not surpass cut-offs, (Sample 1: ΔCFI = -.007, ΔRMSEA = -.010; Sample 2: ΔCFI = -.006, ΔRMSEA = -.014). Overall, the Satisfaction with Life scale demonstrated measurement invariance across groups suggesting that the assessment of life satisfaction did not differ across singles and partnered individuals.

**Sexual Satisfaction*.*** In line with existing work demonstrating a one-factor structure (Park & MacDonald, 2022), we tested a one-factor structure of sexual satisfaction. Firstly, fit indices indicated good fit in both samples, CFI > .95, RMSEA < .08. Model fit indices did not change beyond the cut-offs when constraining the factor loadings to assess metric invariance, (Sample 1: ΔCFI -.001, ΔRMSEA = -.014; Sample 2: ΔCFI = .00, ΔRMSEA = -.006), as well as when additionally constraining intercepts to assess scalar invariance (Sample 1: ΔCFI = .001, ΔRMSEA = -.014; Sample 2: ΔCFI -.001, ΔRMSEA = -.004). Overall, the Sexual Satisfaction scale demonstrated measurement invariance across groups suggesting that the assessment of sexual satisfaction did not differ across singles and partnered individuals.

**References**

Choi, S. W., Gibbons, L. E., & Crane, P. K. (2011). Lordif: An R package for detecting

differential item functioning using iterative hybrid ordinal logistic regression/item

response theory and Monte Carlo simulations. *Journal of Statistical Software*, 39, 1–30.

<https://doi.org/10.18637/jss.v039.i08>

Lehmann, V., Tuinman, M. A., Braeken, J., Vingerhoets, Ad. J. J. M., Sanderman, R., &

Hagedoorn, M. (2015). Satisfaction with Relationship Status: Development of a New

Scale and the Role in Predicting Well-Being. *Journal of Happiness Studies*, *16*(1), 169–

184. <https://doi.org/10.1007/s10902-014-9503-x>

Marsh, H. W., Lüdtke, O., Muthén, B., Asparouhov, T., Morin, A. J. S., Trautwein, U., &

Nagengast, B. (2010). A new look at the big five factor structure through exploratory

structural equation modeling. Psychological Assessment, 22(3), 471–

491. [https://doi.org/10.1037/a0019227](https://psycnet.apa.org/doi/10.1037/a0019227)

Park, Y., MacDonald, G. (2022). Single and Partnered Individuals’ Sexual Satisfaction as a

Function of Sexual Desire and Activities: Results Using a Sexual Satisfaction Scale

Demonstrating Measurement Invariance Across Partnership Status. *Archives of Sexual*

*Behavior*, 51, 547–564 (2022). <https://doi.org/10.1007/s10508-021-02153-y>

**Supplementary Material B: Personality Facet Reliabilities**

**Table S1.** Alpha reliabilities across all 15 facets within the big five personality domains for Sample 1 and Sample 2.

**Supplementary Material C: R Packages Used**

Behrendt S (2023). *lm.beta: Add Standardized Regression Coefficients to Linear-Model-*

*Objects*. R package version 1.7-2, <https://CRAN.R-project.org/package=lm.beta>.

Ben-Shachar, M., Lüdecke, D., Makowski, D. (2020). *effectsize:* *Estimation of*

*Effect Size Indices and Standardized Parameters*. *Journal of Open Source*

*Software*, *5*(56), 2815. <https://doi.org/10.21105/joss.02815>

Long, J. A. (2019). *Interactions: Comprehensive, User-Friendly Toolkit for Probing Interactions*.

R package version 1.1.0, <https://cran.r-project.org/package=interactions>.

Chan, C., Chan, G. C., Leeper, T. J., and Becker, J. (2021). *rio: A Swiss-army knife for data file*

*I/O*. R package version 0.5.29.

Wickham H., Averick M., Bryan J., Chang W., McGowan L. D., François R., Grolemund

G., Hayes A., Henry L., Hester J., Kuhn M., Pedersen T. L., Miller E., Bache S. M., Müller K., Ooms J., Robinson D., Seidel D. P., Spinu V., … Yutani, H. (2019). Welcome to the tidyverse. *Journal of Open Source Software*, *4*(43), 1686.

<https://doi.org/10.21105/joss.01686>

Revelle, W. (2023). *psych: Procedures for Psychological, Psychometric, and Personality*

*Research*. Northwestern University, Evanston, Illinois. R package version 2.3.6,

https://CRAN.R-project.org/package=psych.

Wickham, H. *ggplot2: Elegant Graphics for Data Analysis*. Springer-Verlag New York, 2016.

­Rosseel, Y. (2012). lavaan: An R Package for Structural Equation Modeling. *Journal of*

*Statistical Software*, *48*(2), 1-36. https://doi.org/10.18637/jss.v048.i02

**Supplementary Material D: ANOVA Subfacet Differences**

**Table S2.** Mixed ANOVA results examining personality interactions with relationship status in both sample 1 and sample 2.

*Note.* Only significant two-way interactions that replicated across samples were probed for subfacet differences across relationship status.

**Supplementary Material E: Subfacet correlation matrix**

**Table S3a. Correlations and Descriptive Statistics Between Personality Subfacets in Sample 1.**

**
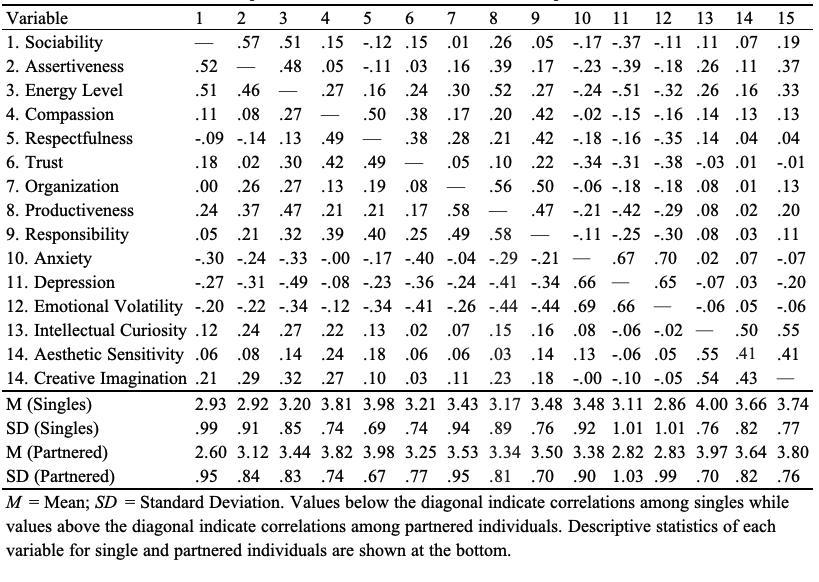
**

**Table 3b. Correlations and Descriptive Statistics Between Personality Subfacets in Sample 2.**

**
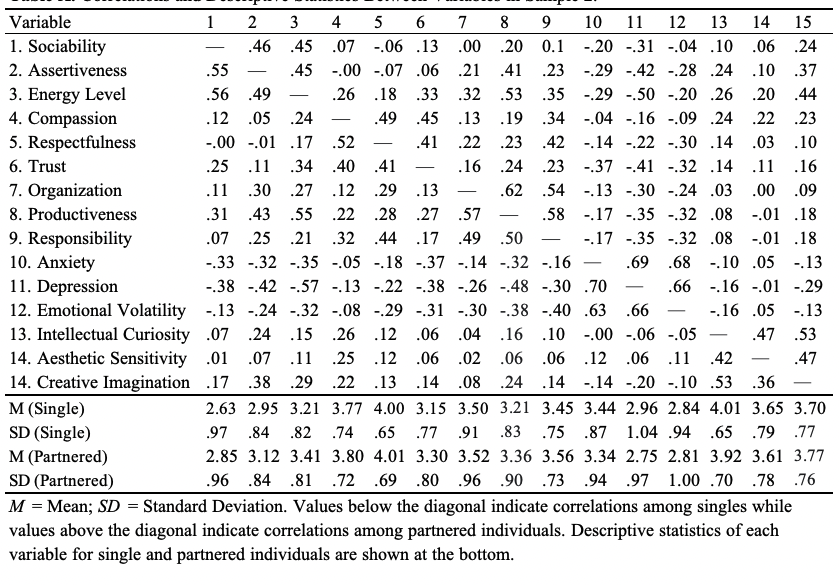
**

**Supplementary Material F: Personality Predictors of Well-being Across Relationship Status**

**Table 4a.** Personality trait and relationship status interactions predicting life satisfaction and sexual satisfaction in Sample 1

| **Sample 1** | **Life Satisfaction** | | | |  | **Sample 1** | **Sexual Satisfaction** | | | |
| --- | --- | --- | --- | --- | --- | --- | --- | --- | --- | --- |
| *β* *SE*  *t* *p* | | | | |  | *β* *SE*  *t* *p* | | | | |
|  |  |  |  |  |  |  |  |  |  |  |
| **IV: Extraversion** | .31 | .09 | 6.72 | <.000 |  | **IV: Extraversion** | .21 | .11 | 4.87 | <.000 |
| Single Status | -.27 | .43 | -1.97 | <.05 |  | Single Status | -.31 | .51 | -2.43 | <.05 |
| Interaction | -.01 | .14 | -0.04 | .97 |  | Interaction | .20 | .16 | -1.61 | .12 |
| **IV: Agreeableness** | .14 | .13 | 3.04 | <.001 |  | **IV: Agreeableness** | .12 | .15 | 2.72 | <.01 |
| Single Status | -.44 | .67 | -2.04 | <.05 |  | Single Status | -.39 | .78 | -1.96 | .05 |
| Interaction | .13 | .18 | .57 | .57 |  | Interaction | -.15 | .21 | -0.75 | .45 |
| **IV: Conscientiousness** | .32 | .10 | 6.86 | <.000 |  | **IV: Conscientiousness** | .15 | .12 | 3.44 | <.000 |
| Single Status | -.13 | .49 | -0.80 | .42 |  | Single Status | -.27 | .59 | -1.87 | .06 |
| Interaction | -.19 | .14 | -1.12 | .26 |  | Interaction | -.26 | .17 | -1.70 | .09 |
| **IV: Neuroticism** | -.41 | .07 | -9.66 | <.000 |  | **IV: Neuroticism** | -.20 | .09 | -4.74 | <.000 |
| Single Status | -.19 | .35 | -1.65 | .10 |  | Single Status | -.60 | .44 | -5.32 | <.000 |
| Interaction | -.11 | .11 | -0.95 | .34 |  | Interaction | .-.08 | .14 | 0.70 | .49 |
| **IV: Openness** | .07 | .12 | 1.39 | .16 |  | **IV: Openness** | .01 | .14 | 0.12 | .90 |
| Single Status | -.35 | .65 | -1.66 | .10 |  | Single Status | -.88 | .75 | -4.64 | <.000 |
| Interaction | .02 | .17 | .11 | .91 |  | Interaction | .35 | .19 | 1.82 | .07 |

**Table 4b.** Personality trait and relationship status interactions predicting life satisfaction and sexual satisfaction in Sample 2

| **Sample 2** | **Life Satisfaction** | | | |  | **Sample 2** | **Sexual Satisfaction** | | | |
| --- | --- | --- | --- | --- | --- | --- | --- | --- | --- | --- |
| *β* *SE*  *t* *p* | | | | |  | *β* *SE*  *t* *p* | | | | |
|  |  |  |  |  |  |  |  |  |  |  |
| **IV: Extraversion** | .32 | .08 | 7.81 | <.000 |  | **IV: Extraversion** | .19 | .11 | 4.96 | <.000 |
| Single Status | -.45 | .37 | -3.61 | <.000 |  | Single Status | -.51 | .48 | -4.28 | <.000 |
| Interaction | .21 | .19 | 1.72 | 0.09 |  | Interaction | .03 | .15 | .23 | .82 |
| **IV: Agreeableness** | .25 | .12 | 6.10 | <.000 |  | **IV: Agreeableness** | .14 | .13 | 3.82 | <.000 |
| Single Status | -.08 | .57 | -0.41 | 0.68 |  | Single Status | -.23 | .71 | -1.29 | .20 |
| Interaction | -.20 | .15 | -1.05 | 0.29 |  | Interaction | -.28 | .19 | -1.56 | .12 |
| **IV: Conscientiousness** | .31 | .08 | 7.94 | <.000 |  | **IV: Conscientiousness** | .14 | .10 | 3.84 | <.000 |
| Single Status | -.33 | .42 | -2.31 | <.05 |  | Single Status | -.49 | .54 | -3.64 | <.000 |
| Interaction | .06 | .12 | 0.44 | .66 |  | Interaction | -.01 | .15 | -0.05 | .96 |
| **IV: Neuroticism** | -.58 | .07 | -8.63 | <.000 |  | **IV: Neuroticism** | -.15 | .09 | -3.91 | <.000 |
| Single Status | .11 | .31 | 0.38 | .71 |  | Single Status | -.37 | .40 | -3.66 | <.000 |
| Interaction | -.30 | .10 | -3.05 | <.001 |  | Interaction | -.14 | .13 | -1.31 | .19 |
| **IV: Openness** | .11 | .10 | 2.68 | <.001 |  | **IV: Openness** | .10 | .13 | 2.62 | <.001 |
| Single Status | -.01 | .59 | -0.06 | .95 |  | Single Status | -.29 | .72 | -1.64 | .10 |
| Interaction | -.29 | .15 | -1.43 | .15 |  | Interaction | -.22 | .19 | -1.23 | .22 |

**Supplementary Material G: Relationship status differences in personality subfacet and well-being associations**

**Table 5a.** Multiple regression subfacet and well-being analyses across relationship status for Sample 1.

Status Satisfaction = Relationship Status Satisfaction.

**Table 5b.** Multiple regression subfacet and well-being analyses across relationship status for Sample 2.

Status Satisfaction = Relationship Status Satisfaction.
